# Access to the US Department of Veterans Affairs health system: self-reported barriers to care among returnees of Operations Enduring Freedom and Iraqi Freedom

**DOI:** 10.1186/1472-6963-13-498

**Published:** 2013-12-01

**Authors:** Christine A Elnitsky, Elena M Andresen, Michael E Clark, Suzanne McGarity, Carmen G Hall, Robert D Kerns

**Affiliations:** 1 School of Nursing, College of Health and Human Services, The University of North Carolina at Charlotte, 9201 University City Blvd, Charlotte, NC 28223, USA; 2 Institute on Development & Disability, Department of Public Health & Preventive Medicine, Oregon Health & Science University, 707 SW Gaines St, Portland, OR 97239, USA; 3 James A. Haley Veterans’ Hospital, Mental Health and Behavioral Science Service, 13000 Bruce B. Downs Blvd, Tampa, FL 33612, USA; 4 Polytrauma and Blast-Related Injuries QUERI, VA Medical Center, One Veterans Drive (152/2E), Minneapolis, MN 55417, USA; 5 PRIME Center (11ACSLG), VA Connecticut Healthcare System, Bldg 35A, 2nd Floor, Room 221, 950 Campbell Avenue, West Haven, CT 06516, USA

**Keywords:** Access to care, Health/psychiatric services, Veterans/psychology, VA health system

## Abstract

**Background:**

The U.S. Department of Veterans Affairs (VA) implemented the Polytrauma System of Care to meet the health care needs of military and veterans with multiple injuries returning from combat operations in Afghanistan and Iraq. Studies are needed to systematically assess barriers to use of comprehensive and exclusive VA healthcare services from the perspective of veterans with polytrauma and with other complex health outcomes following their service in Afghanistan and Iraq. These perspectives can inform policy with regard to the optimal delivery of care to returning veterans.

**Methods:**

We studied combat veterans (n = 359) from two polytrauma rehabilitation centers using structured clinical interviews and qualitative open-ended questions, augmented with data collected from electronic health records. Our outcomes included several measures of exclusive utilization of VA care with our primary exposure as reported access barriers to care.

**Results:**

Nearly two thirds of the veterans reported one or more barriers to their exclusive use of VA healthcare services. These barriers predicted differences in exclusive use of VA healthcare services. Experiencing any barriers doubled the returnees’ odds of not using VA exclusively, the geographic distance to VA barrier resulted in a 7 fold increase in the returnees odds of not using VA, and reporting a wait time barrier doubled the returnee’s odds of not using VA. There were no striking differences in access barriers for veterans with polytrauma compared to other returning veterans, suggesting the barriers may be uniform barriers that predict differences in using the VA exclusively for health care.

**Conclusions:**

This study provides an initial description of utilization of VA polytrauma rehabilitation and other medical care for veteran returnees from all military services who were involved in combat operations in Afghanistan or Iraq. Our findings indicate that these veterans reported important stigmatization and barriers to receiving services exclusively from the VA, including mutable health delivery system factors.

## Background

Since 2002, over 2.3 million Americans have been deployed in support of the wars in Afghanistan or Iraq (commonly called Operation Enduring Freedom and Operation Iraqi Freedom [OEF-OIF]). Of that total, over 1 million returnees have accessed services through the US Department of Veterans Affairs (VA) Veterans Health Administration. The VA is the single largest healthcare provider for this population of veterans. Many veterans of Afghanistan and Iraq have experienced exposure to blasts and explosions, resulting in multiple complex injuries to body systems, emotional distress and mental disorders, [1,2] and pain [3-5]. These multiple injuries to two or more body systems or parts resulting in physical, psychological, cognitive or other psychosocial impairments have been designated “polytrauma” by the VA. Among OEF-OIF war veterans, there also is a high prevalence of Post Traumatic Stress Disorder (PTSD) [1,2,6] which often occurs in tandem with pain [7].

To meet the needs of returning veterans and military with polytraumatic injuries, VA implemented the Polytrauma System of Care which currently is composed of five Polytrauma Rehabilitation Centers (PRC), 23 Polytrauma Network Sites (PNS), and more than 130 support sites with Polytrauma Support Clinical Teams or Points of Contact. PRCs provide comprehensive, acute inpatient rehabilitation, and PNSs provide interdisciplinary, post-acute rehabilitation services [7]. In addition to veterans who are registered with these care centers, the VA created a registry of Veterans returning from Afghanistan and Iraq, called the Operation Enduring Freedom and Operation Iraqi Freedom (OEF-OIF) registry, to provide critical information for concurrent care and consultation across multidisciplinary experts. Both of these veteran tracking sources were used in the present study, combining a broad spectrum of veterans of OEF-OIF including those with designated polytrauma, and others with complex health service needs.

While veterans of both OEF-OIF systems are eligible to use VA healthcare services for several years following their return, they may face barriers to seeking care beyond the cost of health care services. For example, veterans of these wars have reported the stigma of mental illness or being a burden to the system as barriers to seeking care [1,8]. While addressing barriers to use of mental health services in this OEF-OIF population is a priority, it is important to recognize that these veterans have other serious needs requiring the use of a broader range of services, and that they might seek or receive these services outside of the VA. Continuity of quality healthcare and reduced cost are both enhanced from exclusive use of VA healthcare, and understanding why veterans choose or are limited in exclusive VA care is essential.

We found no studies that addressed barriers to use of comprehensive and exclusive VA healthcare services from the perspective of veterans of OEF-OIF with polytrauma. One qualitative study described polytrauma rehabilitation and its impact on providers and identified potential provider-perceived barriers at VA PRCs [9]. Other studies have examined military service member perceptions of barriers to military mental health services [1] or veteran perceptions of barriers and facilitators to mental health care [10,11] and PTSD treatment in particular [8]. Health system barriers to care such as long wait times and long distances to treatment facilities reduce veterans’ ability to access care [12] and this has important implications for discontinuities in utilization of mental health and non mental health services [13]. Thus, a more comprehensive view of access is important to understanding the issue of barriers to care.

**Figure 1:**
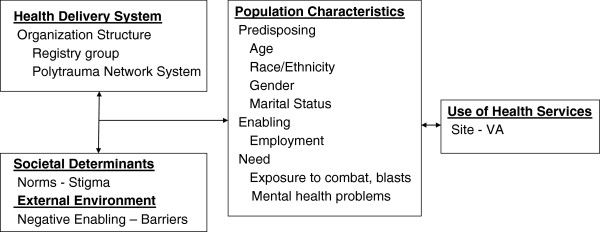
Model of VA service utilization.

Our research question was theory-driven. The Behavioral Model of Health Care Utilization is the leading framework for examining predictors of health care use, and is especially well suited to understanding VA care for OEF-OIF veterans. This model considers health care system, population, and societal and external environment factors that predict health care service use [14,15]. The health care system organization determines a person’s use of services, such as PRC or OEF-OIF Registry [15]. Three sets of population factors contribute to an individual’s propensity to use health care services: predisposing, enabling, or need [15]. ***Predisposing factors*** exist before the onset of injury, such as demographics. ***Enabling resources***, such as income and insurance, impede or enhance access to care. The ***need for services*** includes diagnoses or symptoms. Barriers, factors that make it difficult to receive services, intervene between the delivery system and its utilization [16,17]. While a number of researchers have applied this model to the study of VA health service use in general, and PTSD in particular, we know of no studies examining the utility of this framework to explain the impact of delivery system, population characteristics, and barriers on exclusive use of VA services among veterans with polytrauma. A graphical representation of our adapted operational model is shown in Figure 1. Our approach modeled barriers for veterans using all their health care from the VA among those using any VA care. This approach included attention to variation in practice associated with different geographical regions and different VA organizations.

## Methods

### Setting/sample

Two of four US VA facilities with regional inpatient Polytrauma Rehabilitation Centers (PRCs) participated in this study; the fifth currently designated PRC was not active at the time of this study. PRC sites treat the greatest number and most complex active duty and retired service members seeking or receiving VA healthcare services and serve as primary components of the VA Polytrauma Network System (PNS). The study facilities, one northern and one southeastern, are both large, tertiary care VA hospitals that provide a broad range of medical and mental health care. Both also maintain a comprehensive list (OEF-OIF registry) of current or former service members who have applied for local VA services. Participants we recruited were military personnel deployed during OEF- OIF who were either receiving or had registered for (but might not have used) VA healthcare. Including participants from both locations’ facilities’ increased regional and ethnic heterogeneity of the sample of OEF-OIF returnees and enriched the ability of the analyses to reflect the changing nature of the definition of polytrauma and the veterans experiencing it.

Participants were recruited from either the OEF-OIF registry (Registry group) or the PNS (PNS group) at the two study sites. Eligibility for the study required that all study participants were deployed to Afghanistan or Iraq between October 2001 through September 2010, capable of reading and writing English, and were judged competent by their providers to provide informed consent. OEF-OIF registry participants were randomly selected from facility lists and recruited using a three-stage process of a mailed letter of study introduction, telephone invitation and information for full screening, and a face-to-face consent procedure. PNS participants were recruited directly from the two local polytrauma programs. Participants were eligible for enrollment at any point during their treatment, including hospitalization. In addition to meeting the overall study inclusionary criteria, PRC patients had to: attain a Rancho Los Amigos [12,18] (a scale of cognitive impairment routinely used in PRCs) level of 6 or greater (minimal to moderate impairment) and receive attending physician clearance to participate. Because the cognitive status (thus study eligibility) of PRC residents often improved dramatically during their inpatient rehabilitation stay, study staff reviewed medical records and progress notes periodically to determine if and when they met study inclusionary criteria. A summary of the recruitment process and associated attrition has been presented elsewhere [5]. Because the registry includes many people whose location changed after their initial entry and could not be located within the constraints of this project, and because registry eligibility was fluid when we sampled, we only include people we spoke to directly as the potential respondents. A total of 359 participants (218 Registry; 141 PNS) completed baseline assessments. Participants received a $30 incentive on completion of the baseline assessment session to offset time investments and travel expenses. The University of South Florida Institutional Review Board and the Minneapolis VA Medical Center Subcommittee on Human Studies and each VA Medical Center’s Research and Development committee approved the study prior to recruitment and data collection.

### Measures

As noted, we selected model covariates, applying the Andersen behavioral model of factors influencing utilization [14,19]. Figure 1 provides specific factors included in our study. We measured mental health problems as self-reported in structured clinical interviews. This interview was an expansion of one developed in 2005 to identify pain and emotional symptoms in returning soldiers [20]. Service connection (as a percentage degree of impairment) was reported as recorded in the VA electronic medical record. Service connection indicates whether the returnee was certified as disabled and was eligible for benefits to compensate for disorders incurred or aggravated during military service [21]. Many returnees with psychiatric diagnoses are classified as service connected, meaning they have priority for VA healthcare services and receive financial disability compensation from VA.

We categorized returnees’ psychiatric diagnoses type based on a number of instruments. Diagnoses were established by in-person clinical interviews utilizing the Mini International Neuropsychiatric Interview (M.I.N.I. version 5.0). The M.I.N.I. is a brief, validated, structured clinical interview designed to yield reliable Axis I DSM-IV psychiatric diagnoses. [22] It has been validated against versions of the Structured Clinical Interview for DSM diagnoses (SCID-P) and the Composite International Diagnostic Interview for ICD-10 (CIDI) [22]. We used the M.I.N.I to identify Major Depressive Disorder and PTSD diagnoses. The Center for Epidemiologic Studies-Depression Scale (CES-D) 20-item measure was used to identify depressive symptomatology [23,24]; it complements the M.I.N.I major depressive disorder diagnosis measure used in this study. The Dyadic Adjustment Scale, Short Form (DAS-SF) 7-item measure of marital adjustment and marital quality discriminates between distressed and adjusted relationships and was used as an index of relationship distress in study participants [25].

Utilization of VA care was characterized by several variables. First, participants were asked if they had received pain treatments or mental health treatments from VA in the last 3 months. Following several probes describing those treatments, participants were asked if they were using VA for all medical services (yes, no). Study participants were also asked if they were planning to use VA services, and if not planning to use VA we asked for the reasons (why not). These were recorded as text, and subsequently coded into one of 10 categories, as described below.

In the Behavioral Model, [14] access to care represents an enabling factor influencing utilization and access barriers represent negative enabling factors. We selected qualitative methods to identify a broad range of barriers that were meaningful to the OEF-OIF veteran participants. Barriers to care were derived from one open-ended question asked of veterans, “What might be barriers to receiving care at the VA?” One team member (SG) coded these responses into categories, and two additional investigators independently coded, then reviewed together the resulting codes. Each respondent was asked to identify up to three barriers to care. Responses were coded into ten variables (Barriers) as “present” or “not present”. Each barrier type was coded as present or absent if it appeared in any of the three responses, and we also constructed a variable with a total count of barriers and coded a binary outcome (0, or 1–3 barriers).

### Analyses

We posed primary hypotheses based on gender and by care system (Registry and PNS) using independent t tests for continuous or quasi-continuous data, and Chi-Square tests for categorical data. In these analyses no correction for multiple comparisons was employed as our primary aim was to identify characteristics of the sample of potential interest to readers.

By using theory-based model building, we acknowledged the different VA facilities and were attentive to variation in practice that might exist in different geographic regions. We conducted exploratory regression models examining groups of predictors of barriers to exclusive use of VA care before using these variables in our models of healthcare utilization. There are theoretically three groups of VA veteran health care users, those who exclusively use, those who use some, and those who use none of the available services. Overall, we examined barriers to the exclusive use of VA care (yes/no). Organization of VA care suggested two specific system barriers that we examined in more detail. These two barriers were distance to the facility, and wait times to care. We also constructed a model using a summary variable of specific various factors classified as any barrier compared to no reported barrier. Covariates in all three models were selected from the common key barriers that emerged from descriptive and model building analysis phases [26].

We computed the odds ratio (OR) and 95% confidence intervals (CI) to examine the association of specific, and any barriers with utilization of exclusive use of VA healthcare using binomial logistic regression. Our final models for each individual (and a summary measure) barrier variable include a parsimonious set of covariates. We included age and gender, and then tested and included potential confounders (e.g., employment, mental health) if they produced a meaningful change in the OR for our veteran group of 10% or more. All analyses were conducted using SPSS (version 19).

### Models

Other predictor variables (health system; predisposing, enabling, need characteristics) were tested within groups, then we tested for a parsimonious final model (forcing in hypothesized variables, such as gender and registry) [26].

To examine the extent to which care system and other factors explain the number of barriers experienced to exclusive use of VA care, we conducted a progressive series of linear regressions. For each regression, we entered measures that contributed to use (e.g., system of care, predisposing variables such as age, gender, marital status, and type of service). We excluded variables from multivariate models when the regression coefficient for groups did not change (data available from authors). We then examined the impact on utilization for any barriers and then looked separately at those with variability and sufficient frequency to test in utilization models (i.e., geographic distance, and wait time barriers).

To examine the extent to which barriers to VA care influence actual use of VA health care, we next conducted a series of logistic regressions in which we examined the unique contribution of care system and other factors to VA use. For each logistic regression model, we entered previously documented population characteristics that contributed to use (e.g., age, gender, marital status; enabling factors such as education, employment). Major differences between groups on barriers and the direction of relationship seemed to indicate a relationship to injuries so we added an Anderson model “need” characteristic to the models (depression as measured by the M.I.N.I. or ever had a mental health problem [yes/no]).

## Results

### Descriptive characteristics

Of the 359 participants, 218 (60.7%) were recruited from the local OEF-OIF site registries (Registry) and 141 (39.3%) originated from the two local VA PNS sites. The demographic characteristics of the overall sample and the two VA PNS sites are summarized in Table 1. The average age of participants was relatively high (35.1 years) and ranged from 20 to 66 years. Women constituted 7.9% of the sample, equal to the current US women veteran population [27] but slightly under-representing VA Health Care registration data for OEF and OIF veterans [28]. The ethnic and racial distribution of the sample reflected an overrepresentation of Hispanics (10.6%) when compared with the veteran population (5.9%) and underrepresentation of Blacks (9.5%) when compared with the overall US veteran population (11.4%).[27] The majority of participants were married (52.4%), had completed their service obligations, and were employed full time (61.8%). The average length of deployment to OEF-OIF was approximately 15 months, 80.2% reported they had been exposed to blasts, and 65.2% were receiving VA benefits for service connected conditions.

**Table 1:** Descriptive characteristics of 359 veterans of operation enduring freedom (OEF) or operation Iraqi freedom (OIF) +

**Variables**	**All veterans N = 359**	**PNS + n = 141**	**OEF/OIF + n = 218**
**Predisposing characteristics**			
Mean age (range 20–66) §§§	35.1 years	32.9 years	36.6 years
Gender (% men)	91.1%	92.9%	89.9%
*Marital status*			
Never married	24.2%	26.2%	22.9%
Married	52.4%	48.9%	54.6%
Living as married	6.7%	6.4%	6.9%
Divorced/separated	16.4%	18.4%	15.7%
Mean years of education §§§	14.5 years	13.8 years	14.9 years
*Race/ethnicity*§			
White non-hispanic	77.4%	85.1%	72.4%
Hispanic	10.6%	5.0%	12.3%
Black non-hispanic	9.5%	9.2%	11.5%
Other groups	2.5%	0.7%	3.7%
*Employment*§§§			
Employed	61.8%	58.2%	64.2%
Student	15.9%	15.6%	16.1%
Unemployed looking for work	11.1%	9.9%	11.9%
Other non-working	10.5%	15.9%	6.9%
*Duty status at baseline *§§§			
Active duty	11.7%	20.6%	6.0%
Inactive reserve	10.6%	13.5%	8.7%
Active reserve	20.9%	23.4%	19.3%
Temporary duty release	1.7%	2.8%	0.9%
Completed service	55.2%	39.7%	65.1%
*Service branch (any service in each branch)* §			
Any Army	49.1%	50.3%	48.2%
Any Navy	8.1%	5.7%	9.6%
Any Air Force	8.7%	4.2%	11.5%
Any Marine	11.2%	14.9%	8.8%
Any National Guard	24.3%	26.9%	22.5%
**Enabling characteristics**			
Service connected (self report)	65.2%	73.8%	59.6%
*Exposures*			
OEF/OIF deployment months (range 0–65 months) §	14.6 months	15.9 months	13.8 months
Months since return (range 0–135 months) §§§	42.4 months	34.1 months	47.8 months
Gulf War tours	12.0%	9.3%	13.8%
Exposed to blast §§§	80.2%	94.3%	71.1%
**Need characteristics**			
Ever had a mental health problem (self report) §§§	67.2%	87.9%	53.7%
CESD† scale mean	18.6	23.2	15.5
Dyadic adjustment scale score (mean)	22.4	21.4	23.1
PTSD diagnosis (y/n) §§§	26.5%	44.7%	14.7%
Major depressive disorder diagnosis §§§	29.5%	42.6%	21.1%
**VA health system geographic location** §			
Southeast	40.9%	28.4%	49%
North	59.1%	71.6%	51%
**Barriers to VA care**			
Any perceived barrier	62.4%	63.8%	61.5%
Wait times	26.7%	23.4%	28.9%
Staff concerns/reputation	15.0%	16.3%	14.2%
Fear/embarrassment/stigma	13.9%	17.0%	11.9%
Distance/location	12.0%	12.8%	11.5%
Paperwork/hassle	10.3%	9.9%	10.6%
Lack of services information	9.5%	10.6%	8.7%
Limited services hours	3.3%	5.0%	2.3%
Other insurance/money/ private MD	3.3%	1.4%	4.6%
Fear military records access	1.7%	2.8%	0.9%
Active duty	1.4%	2.8%	0.5%
Total barriers (0–3) mean ± standard deviation (SD) Among all veterans, 0 barriers = 37.6%; 1 = 28.1%; 2 = 18.3%; 3 = 15.8%	1.1 (± 1.1)	1.2 (± 1.1)	1.1 (± 1.1)
**Utilization**			
VA current treatment §§§	53.0%	65.9%	40.3%
VA pain treatment last 3 months §§§	37.8%	52.5%	28.2%
VA mental health treatment last 3 months §§	51.6%	61.4%	41.7%
All VA services in last 3 months §§§	53.2%	67.6%	44.0%
Community pain treatment received last 3 months	23.6%	24.6%	23.0%
Community mental health treatment 3 months	15.3%	16.9%	13.6%
In- patient §§§	15.1%	28.4%	6.5%
VA treatment received last 3 months §§§	61.8%	47.5%	71.1%
Community treatment received last 3 months §§§	40.9%	26.2%	50.5%

+ OEF-OIF returnees from PNS is Polytrauma Network System; other OEF/OIF returnees from Registry.

§ p < 0.05§§ p < 0.01 §§§ p < 0.001 Tests between PNS and OEF/OIF groups (means, percentages).

† Center for Epidemiologic Studies Depression (20 item) scale.

Barriers to VA care are reported in Table 1. The majority of participants (62.4%) reported concerns about stigmatization and at least one barrier to VA care. Barriers to VA care reported by participants and categorized by investigators included: (a) Wait times (26.7%); (b) concerns about staff /reputation for care (15%); (c) fear/embarrassment/stigma (13.9%); (d) distance/location (12%); (e) paper work/hassle (10.3%); (f) lack of information about services (9.5%); (g) limited hours for services (3.3%); (h) veteran had other insurance/monetary support/or private doctor (3.3%); (i) fear of military accessing health records (1.7%); and (j) on active duty (1.4%). Both VA care groups generally perceived an equivalent average number of barriers (about 1.1 or 1.2 barriers). For both groups of participants, the most likely barrier to VA care was wait times; the least reported barriers and stigma were having other insurance for PNS participants, and for the OEF-OIF registry group, active duty status and fear of the military accessing their health records. Wait times, reported by participants in open-ended barrier items, included a variety of types, for example, “wait times for appointments are very long”, or “get seen quicker in private facility”. Distance/ location barriers were described in terms of needing to be “closer to home,” or “not being close by- 45 minutes away.”

There were minor differences in the barriers and stigma we tested and none were statistically significant. While we were concentrating on the experience of three dichotomous categories of any barrier plus the two primary system barriers, we conducted ad hoc exploratory analyses of outcomes with models including other specific barriers that are descriptively shown in Table 1.

As the utilization characteristics indicate, a slight majority of participants (53%) were currently receiving VA treatment. PNS participants were more likely to have received pain treatment (52.5%), mental health treatment (61.4%), or all health services (67.6%) from VA in the last 3 months than OEF/OIF registry participants.

The multivariate logistic regression models provided more detailed results about the association between two specific barriers among OEF-OIF returnees and exclusive use of VA for their care. As noted above, we focused on three barriers in the multivariate models: we examined the presence of ***any*** reported barrier, and then two specific VA health delivery system barriers; wait times, and distance location. Table 2 presents the summary of final models of each of the multivariate models of barriers. Because the three models have similar estimates, we provide only the final model in our results section. The individual model constructions with progressive additions of blocks of variables based on the Andersen model are available on line as Additional files 1, 2 and 3.

**Table 2:** Summary of multivariable analyses of barriers to exclusive VA care among OEF-OIF veterans

**Model**	**Odds ratio (OR) and 95% Confidence intervals (CI)**		
	**Individual main effect models**		
	Any barrier*	Distance or location barrier*	Wait times barrier*
Recruitment Group + PNS compared to OEF/OIF registry	0.54 (0.31, 0.93) §	0.52 (0.30, 0.90) §	0.56 (0.33, 0.96) §
North compared to Southeast	1.11 (0.67, 1.86)	1.05 (0.63, 1.77)	1.19 (0.72, 1.99)
Age per year	1.02 (0.99, 1.05)	1.01 (0.98, 1.04)	1.02 (0.99, 1.05)
Women compared to men	1.04 (0.43, 2.51)	1.10 (0.43, 2.72)	1.03 (0.44, 2.46)
Married compared to not	0.59 (0.35, 1.00)	0.47 (0.27, 0.82) §§	0.55 (0.33, 0.94) §
*Employment:*			
Employed (Full or part time)	1.00 (Reference)	1.00 (Reference)	1.00 (Reference)
Student	0.31 (0.15, 0.65) §§	0.30 (0.14, 0.65) §§	0.29 (0.14, 0.60) §§§
Unemployed looking for work	0.75( 0.35, 1.59)	0.73( 0.34, 1.56)	0.71( 0.34, 1.50)
Other non-working	0.14 (0.05, 0.39) §§§	0.11 (0.4, 0.35) §§§	0.13 (0.05, 0.37) §§§
Major Depressive Disorder diagnosis	0.68 (0.38, 1.20)	0.73 (0.40, 1.33)	0.66 (0.37, 1.16)
Ever had a mental health problem	2.07 (1.18, 3.62) §	2.17 (1.23, 3.83) §§	1.86 (1.06, 3.25) §
Experienced **Any Barrier** §§	2.18 (1.32, 3.59) §§		
Experienced **Distance Barrier** §§§		7.29 (3.01, 17.67) §§§	
Experienced **Wait Time Barrier**§			2.07 (1.17, 3.68) §

+ PNS is the Polytrauma Network System; other OEF-OIF veterans from Registry.

§ p < 0.05§§ p < 0.01 §§§ p < 0.001.

*See Additional file 1 for progressive entry of sets of variables for the Any Barrier main effects model.

*See Additional file 2 for progressive entry of sets of variables for the Distance Barrier main effects model.

*See Additional file 3 for progressive entry of sets of variables for the Wait Times Barrier main effects model.

As shown in Table 2, we report three models, one on each of the major barriers (i.e., any barriers, distance or location barrier, and wait time barrier). All three major barriers maintained significant associations with the exclusive use of VA care outcome, after accounting for previously documented health delivery system and population characteristics. In the final model examining the relationship with any barrier we found Veterans who experienced ***any*** barriers had double the odds of not using VA care exclusively. Veterans who experienced distance or location barriers, that is how far they are from a VA facility, had 7- fold increased odds of not using VA care exclusively. Veterans who experienced wait time barriers had double the odds of not using VA care exclusively.

## Discussion

This study assessed barriers to use of exclusive VA healthcare services among veterans of OEF and OIF who were on active duty or discharged military personnel of all military services who were either receiving or registered for VA health care. However, we found almost two thirds reported one or more barriers to receiving VA care. Both the OEF-OIF and the PNS groups described barriers that hindered receiving exclusive VA care. These veterans reported that barriers included wait times, distance to the VA facility, concerns about VA staff reputation, paperwork hassle, lack of information, limited service hours, fear/embarrassment/stigma, and having other insurance.

Both those in the OEF-OIF and the PNS groups reported fear/embarrassment/stigma. For example, participants reported embarrassment and concern associated with using VA services, such as “being a burden to the system,” perceiving this as “welfare,” or thinking they “don’t deserve it,” and that “other people need it more,” or “feeling embarrassed [because] older veterans need it more.”

We found that absence of these reported barriers predicted exclusive use of VA healthcare services from the perspective of these U.S. OEF-OIF returnees. Experiencing any barriers doubled the returnees’ odds of not using VA, the distance to VA barrier resulted in a 7 fold increase in the returnees’ odds of not using VA, and the wait time barrier doubled the returnees’ odds of not using VA. This analysis shows associations in this cross sectional study about barriers that are consistent with other reports. However, given this mounting evidence an interventional study design would be needed to see whether or not removing these barriers would increase access and use of VA care.

Selected barriers and stigma (e.g., military record access, being active duty) in this sample of participants from all military services are similar to those of active duty Army and Marine returnees [1]. Participants in the current study reported having other insurance and funds to see private care providers outside of VA (3.3%) as a barrier to exclusive use of VA services. While the Hoge [1] sample reported concerns about the costs of care (10-25%), participants in the current study did not report costs as a barrier. While the current sample described distance to a VA facility as a barrier, the Hoge [1] sample saw the barrier to service utilization as a transportation issue.

We placed barriers and stigma within the Andersen behavioral model of service use to explain the impact of delivery system, population characteristics, and external environment and societal factors on utilization of VA services in this population of returnees while attending to variation in practice in different geographical regions and VA organizations. External environment determinants included any barriers and the specific barriers of distance and wait times. Societal determinants included three stigma, fear/embarrassment, access to military records, and being active duty [14].

The Behavioral Model of health care utilization and the barriers and facilitators that our returnees reported suggest targets for intervention. Findings indicate 2 specific areas that warrant attention: 1) wait times; and 2) distance and location of VA services. As participants described in their own words, “wait times for appointments are very long,” “long wait in ER,” “get seen quicker in private facility,” “pharmacy takes too long”. Wait times are a commonly reported barrier of health care systems [29].

Distance to a facility, a well-known barrier, was identified **e**ven among these active and retired military, who have access to care during active service and following retirement, given their benefits. Distance to VA facilities has previously been studied, using informatics approaches as geographic information system (GIS) tools to map VA patients and their access to specialty care [30,31]. However, these studies have not included a focus on the OEF-OIF population [32]. While 5 PRCs and numerous other levels of the VA polytrauma system of care were implemented, it is not clear if travel bands to the nearest VA facility with polytrauma specialty care clinics were developed in the original planning.

Access barriers in these veteran groups could have a wide range of negative effects on service utilization and outcomes. The findings highlight areas where VA decision makers may act to enhance access to care that is available to OEF-OIF returnees by targeting distance and wait time barriers that are particularly salient among this population. Such efforts will ultimately contribute to maximizing exclusive use of VA among OEF-OIF returnees.

A major advantage of this study is its ability to provide insight into the experiences of a sample of OEF-OIF returnees from two regions of the country. However the sample accepting our invitation to participate in the study is not necessarily representative of all returnees. Due to limitations in our sampling and tracking within sampling frames, the results may not represent all veterans using VA. If OEF-OIF returnees who were less satisfied with their care were more likely to participate in the study, then the results may overestimate barriers to use of VA care for all services. Another limitation is that qualitative data describe up to 3 different barriers per veteran. While the majority listed 0–1 barriers, and only 57 participants listed 3 barriers, it is possible we have undercounted the barriers and other difference might emerge in future studies and different veteran samples. While we were able to gain insights into a variety of important stigma and barriers to VA care, studies that can assess barriers to care of OEF-OIF returnees who do not use VA services are also needed.

Many returnees may not seek needed mental health care due to public stigma and personal fear and embarassment that constitute barriers to using VA care [33]. Forty-five percent of the current study’s sample was still on active, reserve, or temporary duty release from the military services. Unique cultural factors contributing to perceived stigma and other barriers, such as fear/embarassment/stigma reported by 14% of participants, present unique challenges for health care systems and providers. As these participants stated, they saw their use of services as a “burden to the system” or perceived it as taking “welfare” from the public while their military role has been to protect the public. These participants stated they “do not deserve” the service while “other people need it more.” Cultural awareness of this population can inform strategies to retain OEF-OIF returnees in exclusive VA health care [34].

Access barriers are highly actionable factors. Efforts to address barriers to care in VA should include greater emphasis on the problems of wait times and distance to facilities, as well as fear/embarrassment/stigma and other barriers to using VA services for all care. Reducing these barriers among OEF-OIF returnees is a priority for policymakers, researchers, clinicians, and leaders who are involved in providing care to these service members who have borne the battle.

## Conclusions

In the current study of returnees who were deployed during OEF-OIF and have access to care as a function of their U.S. military and VA benefits, we still found a wide variety of system barriers, the most salient of which were wait times and distance to their VA facility. This study broadens our knowledge of use of VA services among OEF-OIF returnees by including a wide array of explanatory factors, and barriers and stigma self-reported by active duty and retired military personnel. Our findings expand the Andersen behavioral model of utilization by explicitly incorporating U.S. returnees’ self-identified barriers into the conceptual framework to explain the impact of population characteristics and external barriers on exclusive use of one national healthcare delivery system organization’s services among veterans with polytrauma.

## Abbreviations

CI: Confidence intervals; CIDI: Composite international diagnostic interview for ICD-10; CES-D: Center for epidemiologic studies-depression scale; DAS-SF: Dyadic adjustment scale, short form; ER: Emergency room; GIS: Global information system; M.I.N.I: Mini international neuropsychiatric interview; OR: Odds ratio; OEF: Operation enduring freedom; OIF: Operation Iraqi Freedom; PNS: Polytrauma network sites; PRC: Polytrauma rehabilitation centers; PTSD: Post traumatic stress disorder; SCID-P: Structured clinical interview for DSM diagnoses; VA US: Department of Veterans Affairs.

## Competing interests

The authors declare that they have no competing interests.

## Authors’ contributions

Study concept and design: CE, EA, MC. Acquisition of data: SM, CH. Analysis and interpretation of data: CE, MC, EA, SM, CH. Statistical analysis: EA, CE. Drafting of manuscript: CE. Critical revision of manuscript for important intellectual content: CE, EA, CH, MC, RK. Obtained funding: MC. Study supervision: MC, CE. Administrative, technical, or material support: CN. All authors read and approved the final manuscript.

## Authors’ information

During this study, CE was affiliated with the Department of Veterans Affairs; she is now affiliated with the University of North Carolina at Charlotte.

During this study EMA was affiliated with the University of Florida; she is now affiliated with Oregon Health & Science University.

## Pre-publication history

The pre-publication history for this paper can be accessed here:

http://www.biomedcentral.com/1472-6963/13/498/prepub

## Supplementary Material

Additional file 1: Table S1 Odds of Not Exclusively Using VA Care among OEF-OIF Veterans Associated with Any Care Barriers. Click here for file

Additional file 2: Table S2 Odds of Not Exclusively Using VA Care among OEF-OIF Veterans Associated with Care Location Barriers. Click here for file

Additional file 3: Table S3 Odds of Not Exclusively Using VA Care among OEF-OIF Veterans Associated with Waiting Time Barriers. Click here for file
