# Supplementary material for: Access to the US Department of Veterans Affairs health system: self-reported barriers to care among returnees of Operations Enduring Freedom and Iraqi Freedom
Source: BMC Health Serv Res. 2013 Dec 1;13:498. doi: 10.1186/1472-6963-13-498 (PMC3893594; doi:10.1186/1472-6963-13-498)
Supplement: Additional file 2: Table S2 — Odds of Not Exclusively Using VA Care among OEF-OIF Veterans Associated with Care Location Barriers. [file 1472-6963-13-498-S2.docx]

**Additional file 2: Table S2:** Odds of Not Exclusively Using VA Care among OEF-OIF Veterans Associated with Care Location Barriers.

| **Sequential Models for Distance Barrier & Exclusive VA Care** | **Odds Ratio (OR) and 95% Confidence Intervals (CI)** | | | |
| --- | --- | --- | --- | --- |
|  | **Model 1*** | **Model 2**** | **Model 3 ***** | **Model 4 ****** |
| Recruitment Group +  PNS compared to OEF/OIF registry | 0.36 (0.23, 0.56) §§§ | 0.41 (0.25, 0.67) §§§ | 0.54 (0.32, 0.92) § | 0.52 (0.30, 0.90) § |
| North compared to Southeast | 1.29 (0.82, 2.01) | 1.33 (0.82, 2.15) | 1.20 (0.72, 1.99) | 1.05 (0.63, 1.77) |
| Age per year |  | 1.02 (0.99, 1.05) | 1.02 (0.99, 1.04) | 1.01 (0.98, 1.04) |
| Women compared to men |  | 1.01 (0.43, 2.33) | 1.07 (0.45, 2.51) | 1.10 (0.43, 2.72) |
| Married compared to not |  | 0.59 (0.35, 0.98) § | 0.56 (0.34, 0.95) § | 0.47 (0.27, 0.82) §§ |
| Employment:  Employed (Full or part time)  Student  Unemployed looking for work  Other non-working |  | 1.00 (reference)  0.33 (0.16, 0.67) §§  0.59 (0.29, 1.22)  0.13 (0.50, 0.35) §§§ | 1.00 (reference)  0.32 (0.15, 0.66) §§  0.69 (0.33, 1.44)  0.17 (0.06, 0.45) §§§ | 1.00 (Reference)  0.30 (0.14, 0.65) §§  0.73( 0.34, 1.56)  0.11 (0.4, 0.35) §§§ |
| Major Depressive Disorder diagnosis |  |  | 0.69 (0.39, 1.21) | 0.73 (0.40, 1.33) |
| Ever had a mental health problem |  |  | 2.00 (1.15, 3.47) § | 2.17 (1.23, 3.83) §§ |
| **Experienced** D**istance Barrier-** §§§ |  |  |  | **7.29 (3.01, 17.67)** §§§ |

+ PNS is the Polytrauma Network System; other OEF-OIF veterans from Registry

§ p<0.05§§ p<0.01 §§§ p< 0.001

***Model 1 includes the health system characteristics**

****Model 2 includes the health system, and participants’ predisposing and enabling characteristics**

*****Model 3 includes the health system, and participants’ predisposing, enabling, and need characteristics**

******Model 4 includes the health system, and participants’ characteristics, and barriers to care variables**
